# The Medical Standpoint

**Published:** 1921-01-01

**Authors:** 


### THE MEDICAL STANDPOINT.

Let us now turn to the second great question in the hospital world—namely, that concerning the medical profession and its work. Although, the more critical of our medical readers may be inclined to feel that relatively little has been actually accomplished towards clearing up the multitudinous institutional difficulties, yet there is little doubt that gradually evidence is being brought to light and collected in such a way that the near future should certainly see the beginning of really practical reconstruction.

As far as the medical and surgical staffs of the existing voluntary hospitals and the leaders of the British Medical Association are concerned, last week's meeting seems to have put it perfectly plainly that the voluntary principle should be maintained and amplified rather than scrapped as a method in keeping only with mid-Victorian times. Whether this meeting was representative of the medical profession as a whole or not, the resolutions carried are a valuable expression of a high standard of mature medical opinion, and, as such, must be carefully considered by the State authorities. Also it gave a definite statement of a policy, and has therefore the advantage of clearing the medico-political atmosphere to a valuable extent.

It is unnecessary to reiterate our own views on the advisability of giving the voluntary system its fair trial under post-war conditions. On every hand one sees change and deep-seated modification of practically every economic condition in the country and until this phase is past, giving a definite indication of what the features of Great Britain's next settled period are likely to be, both the State and the medical profession would undoubtedly be singularly ill advised to throw over an institutional system which has done such magnificent work in the past. Let the doctors and laymen alike first try to improve it, make it more elastic and ultimately better adapted to modern times. If all concerned co-operate wholeheartedly and in that spirit which has always been associated with hospital work in general, then we should soon see light breaking in the present darkness. Personal service and the forgetting of self-interest in pursuit of a great national ideal are outstanding needs of the moment, whether we turn to the individual, the professional societies, or the masses. A New Year has rarely begun when the call was so clear or the opportunities greater.

But a few hours ago we had the good fortune to be among the Christmas celebrations of one of our greater hospitals. If we can so use the term, that hospital was thronging with life in some of its most beautiful phases. It is almost impossible to describe the atmosphere of such a Hospital Christmas, but it embodies and illustrates in a thousand ways many of those lessons of which the nation seems so badly in need. Many a pain and worry are forgotten in our hospitals at these times, but how much greater is the moral taught to him who is not a patient, how wonderful, if but for one moment, to forget self! May we hope that there has this Christmas been a record stream of visitors to our hospital wards? If so, some at least of our multitudes will have learned a lesson to be remembered their lives long.

It may seem a long cry from a children's Christmas-tree and concerts in a hospital ward to the formalities of the medico-political world. But we contrast and compare them because they are, or should be, pervaded by the same spirit of ungrudging service, and have in the past been guided by the same professional ideals. Cannot medical men as a profession return in full measure to the pre-war ideals of voluntaryism and tackle their problems of reconstruction as a united whole, and not as a camp divided against itself, and that seemingly through reasons of self-interest?

